# Role of Neutrophils and NETs in Animal Models of Thrombosis

**DOI:** 10.3390/ijms23031411

**Published:** 2022-01-26

**Authors:** Estelle Carminita, Lydie Crescence, Laurence Panicot-Dubois, Christophe Dubois

**Affiliations:** 1 Aix Marseille Univ, INSERM 1263 (Institut National de la Santé et de la Recherche), INRAE 1260 (Institut National de la Recherche Agronomique et de l’Environnement), C2VN (Center for CardioVascular and Nutrition Research), 13885 Marseille, France; estelle.carminita@univ-amu.fr (E.C.); Lydie.crescence@univ-amu.fr (L.C.); christophe.dubois@univ-amu.fr (C.D.); 2 Aix Marseille University, PIVMI (Plateforme d’Imagerie Vasculaire et de Microscopie Intravitale), C2VN (Center for CardioVascular and Nutrition Research), 13385 Marseille, France

**Keywords:** in vivo, thrombosis models, endothelium, platelets, neutrophils, NETs

## Abstract

Thrombosis is one of the major causes of mortality worldwide. Notably, it is not only implicated in cardiovascular diseases, such as myocardial infarction (MI), stroke, and pulmonary embolism (PE), but also in cancers. Understanding the cellular and molecular mechanisms involved in platelet thrombus formation is a major challenge for scientists today. For this purpose, new imaging technologies (such as confocal intravital microscopy, electron microscopy, holotomography, etc.) coupled with animal models of thrombosis (mouse, rat, rabbit, etc.) allow a better overview of this complex physiopathological process. Each of the cellular components is known to participate, including the subendothelial matrix, the endothelium, platelets, circulating cells, and, notably, neutrophils. Initially known as immune cells, neutrophils have been considered to be part of the landscape of thrombosis for more than a decade. They participate in this biological process through their expression of tissue factor (TF) and protein disulfide isomerase (PDI). Moreover, highly activated neutrophils are described as being able to release their DNA and thus form chromatin networks known as “neutrophil extracellular traps” (NETs). Initially, described as “dead sacrifices for a good cause” that prevent the dissemination of bacteria in the body, NETs have also been studied in several human pathologies, such as cardiovascular and respiratory diseases. Many articles suggest that they are involved in platelet thrombus formation and the activation of the coagulation cascade. This review presents the models of thrombosis in which neutrophils and NETs are involved and describes their mechanisms of action. We have even highlighted the medical diagnostic advances related to this research.

## 1. Introduction

Hemostasis is a physiological process of host defense that acts to stop bleeding in case of injury to keep the blood flow contained and under pressure. The cellular and molecular phenomenon associated with hemostasis is coagulation. Thrombosis is the pathological process that results from hemostasis [1]. It is associated with many pathologies, such as myocardial infarction, stroke, pulmonary and cerebral embolism, phlebitis of the lower limbs, and thrombosis associated with cancer. It is therefore one of the major causes of death in the world. Thrombus formation can be triggered by different mechanisms, mainly involving the subendothelium, endothelium, platelets, neutrophils [2] and, more recently, neutrophils extracellular traps (NETs) [3].

Neutrophils are a subpopulation of leukocytes known to play a major role in initial immunity [4]. Indeed, neutrophils are the first cells to migrate to the site of infection, or at least of recognized danger, by the organism [5]. Neutrophils have several defense mechanisms. The best-known mechanism, which is shared with monocytes, is phagocytosis. Phagocytosis is a biological process in which, once the neutrophil has recognized, for example, a bacterium, it engulfs, digests, and destroys the bacterium; these actions are driven by the bactericidal proteins contained within its granules. The second mechanism is simply degranulation, i.e., the secretion of proteins. Once activated by the recognition of a danger, the neutrophil is able to degranulate, i.e., to release its surface molecular components initially contained in its granules. Some of these molecular components, such as neutrophil elastase, cathepsin G, and myeloperoxidase, have bactericidal and toxic properties. Finally, the last and most recent mechanism described is the production of NETs [3]. In the case of excess stimulation, neutrophils undergo important intracellular changes. They mix their deoxyribonucleic acid (DNA) with their granular content and expel this ensemble into the extracellular environment. This chromatin decorated with neutrophilic serine proteases then serves as an adhesive support with the aim of trapping bacteria and thus limiting their dissemination in the body [6].

In 2012 [7], our team showed that following laser beam injury, neutrophils were the first cells present at the site of thrombosis, even before platelets. Activated neutrophils express tissue factor (TF) on their surface and generate the production of thrombin. Thrombin then activates platelets, which form platelet thrombi and generate fibrin. In this model, the procoagulant activity appears to come from the presence of TF on the surface of neutrophils. Several publications, such as von Bruhl et al. [8], Brill et al. [9], and Fuchs et al. [10], have confirmed the role of neutrophils in thrombus formation and the activation of the coagulation cascade in other thrombosis models, including the deep vein thrombosis (DVT) model.

Moreover, Bassuk et al. demonstrated by immunofluorescence and western blot that the expression of protein disulfide isomerase (PDI) is critical for the activation of tissue factor in neutrophils, which confers double procoagulant activity [11]. The authors added that by using RNA hybridization and Northern blot experiments, they found that neutrophils were able to code directly for the PDI gene. Thus, neutrophils play an important role in the activation of the coagulation system in this model.

Finally, serine proteases in neutrophils are thought to have the ability to hydrolyze the tissue factor pathway inhibitor molecule (TFPI), and thus inhibit the extrinsic pathway of activation of the coagulation cascade [12]. The mechanism proposed by the authors is that: (1) TFPI circulating and/or released by cellular actors, such as the endothelium and platelets, is recruited to the site of injury and then (2) incorporated into the forming thrombus, where (3) its degradation by serine proteases from the neutrophils present can take place. In vivo, the predominant beta isoform of TFPI would probably be degraded.

Neutrophils can also activate the coagulation cascade through the production of NETs. NETs are filamentous structures consisting of DNA and serine proteases formerly contained in the numerous granules of neutrophils. They are released following the strong activation of neutrophils. NETs were first described by Brinkman et al. in 2004 following a Staphylococcus aureus infection [6]. Other bacterial stimuli (Streptococcus pneumoniae [13], Escherichia coli [14], etc.) or chemical agonists (calcium ionophore A23187, phorbol myristate acetate (PMA) [15], etc.) are also able to induce NETs formation (NETosis) in vitro. NETosis is described through several steps: (1) the hyperactivation of neutrophils; (2) the clustering of nuclear content with granular content; (3) the decondensation of chromatin and rupture of the nuclear membrane; and finally, (4) the rupture of the plasma membrane and the release of NETs [3]. The NETosis phenomenon thus described has since been extensively studied in humans, animal models, and numerous pathologies. Indeed, in addition to microbial control, NETs have been described as involved not only in cancers but also in various inflammatory phenomena, such as lung damage, or in certain autoimmune diseases, such as vasculitis. Among other pathologies, cardiovascular diseases and the thrombosis phenomenon often associated with them have also raised questions among scientists. Recently, the involvement of NETs has been described in thrombosis. In vivo studies show that in mouse models of DVT, “specific” markers of NETs are present in thrombi, including extracellular DNA, myeloperoxidase (MPO), citrullinated histones H3 (CitH3) and H2, and neutrophil elastase (NE) [10].

Thus, according to the literature, neutrophils and NETs may be important cellular and molecular actors involved in the activation of the coagulation cascade leading to the generation of a stable platelet thrombus.

Many methods are commonly used to experimentally initiate thrombosis for in vivo animal models. All of these models are used to better understand the cellular and molecular mechanisms involved in the pathogenesis of thrombus formation. Some types of injury are known to induce thrombosis after denudation of the endothelium, a mechanism notably found in atherosclerotic plaque rupture and aneurysms. Other models preserve the integrity of the endothelium and activate it. This mechanism is present in human pathologies such as lower limb phlebitis and cancer-associated thrombosis, and in immunothrombotic diseases, including infections.

The study of these numerous pathophysiological mechanisms allows the development of new therapeutic strategies to fight cardiovascular mortality worldwide.

## 2. Neutrophils

Neutrophils are blood cells belonging to the white lineage. They are granulocyte-like leukocytes that play a major role in immunity, and represent the most abundant population of immune cells in human blood. Neutrophils are the first cells or effectors recruited to the inflammation site and thus represent the first line of host defense. Neutrophils act through several mechanisms to fight potential infections: (1) phagocytosis; (2) the secretion of granules containing cytokines, chemokines, and anti-inflammatory molecules; (3) the generation of reactive oxygen species (ROS); and, finally, (4) the formation of neutrophil extracellular traps (NETs).

Initially, neutrophils are formed in the bone marrow and remain there for approximately five to seven days. In the case of infection, neutrophils migrate to the infected target organ and act as immune cells. However, in the absence of infection, neutrophils are released from the bone marrow into the general blood circulation, where they patrol for six to nine hours and then migrate to the liver and spleen for elimination [4].

Neutrophils are cells with a polylobed nucleus segmented into two to five lobes with dense chromatin. The cytoplasm is clear and has numerous granules. They are cells of approximately fifteen microns in humans and seven microns in mice [16]. Upon activation and depending on the stimulus, the neutrophil releases its granular content with, in this order: (1) secretory granules containing receptors for several interleukins (IL 1, 4, 6, 10, 13, 17, and 18), interferons, and complement (C1qR); (2) tertiary gelatinase-rich, argininase-1, and cytochrome b558; (3) secondary or “specific” releasing receptors for urokinase (uPAR), tumor necrosis factor (TNF-R), and fibronectin (fibronectin-R); and, finally, (4) primary or “azurophilic” containing serine proteases such as neutrophil elastase (NE), myeloperoxidase (MPO), and cathepsin G [17,18].

For several years, neutrophils have been of great interest in human pathologies [19] other than infectious ones. They are involved in chronic inflammatory diseases (systemic lupus erythematosus, psoriasis, rheumatoid arthritis), cancers (leukemia, lung and pancreatic ductal adenocarcinoma, head/neck and colorectal cancer, hepatocellular carcinoma), and thrombotic cardiovascular diseases (acute stroke, myocardial infarction, lower limb phlebitis).

Neutrophils, similar to macrophages, are a heterogeneous cell population divided into several subpopulations. Two subpopulations tend to be known: a so-called “normal” subpopulation, also named N1 or normal density neutrophils (NDN), which is in the majority, and a N2 or low-density neutrophils (LDN) subpopulation in the minority. The existence of these two subpopulations of neutrophils has been described in cancer, where the N2 or LDN subpopulation of neutrophils becomes the majority and presents properties that promote tumor growth and metastatic dissemination [20].

## 3. Neutrophils Extracellular Traps (NETs)

NETs are DNA fibers expelled by neutrophils that have undergone strong activation. They were first described in 2004 by Brinkman and colleagues following an incubation of neutrophils with a bacterial pathogen such as Staphylococcus aureus [6].

The process leading to the formation of NETs is called NETosis and is described as a four-step biological phenomenon beginning with (1) a strong activation of neutrophils, leading to (2) the translocation of granular content to nuclear content, followed by (3) DNA decondensation and membrane disruption, and finally to (4) the expulsion of DNA fibers into the extracellular environment [3]. The mechanisms of NETs formation involve NADPH oxidase, ROS generation, require the key enzyme peptidyl arginine deaminase (PAD4 4) responsible of the histone citrullination [21].

The primary role of NETs is immune. Like neutrophils, NETs act as barriers to pathogens and are therefore part of the body’s defense system par excellence. It was described that the DNA fibers that make up NETs are highly adhesive and carry bactericidal molecules such as lactoferrin, LL37 peptide, and proteinase 3, thus limiting the dissemination of pathogens in the body and ensuring their death/destruction [22].

In vitro, several agonists can be used to induce NETosis. Classically, PMA, calcium ionophore, LPS, and many bacteria (Streptococcus pneumoniae, Streptococcus pyogenes, Staphylococcus aureus, Escherichia coli, Salmonella typhimurium, or Shigella flexnerii) can form NETs from purified quiescent neutrophils within 30 min to several hours (3–4 h). The concentration of agonists used and the number of neutrophils undergoing activation are two parameters that affect the time required for NET formation [23].

Morphologically, electron microscopy experiments in particular allow the characterization of neutrophils entering in NETosis with disrupted nuclear and plasma membranes, a clear cytoplasm with granules approaching the center of the cell close to the nucleus and finally extracellular DNA fibers [24].

Regarding the protein profile of these structures, both in vitro [24] and in vivo [8] studies show that NETs express several proteins originally contained in neutrophilic granules such as serine proteases NE, MPO, and cathepsin G. In an original way, NETs show a significant citrullination of its histones (2, 3, and 4). CitH3 was used as a NETs reference marker.

Like neutrophils and since their discovery, NETs have been of great interest in the understanding of human pathologies. Considered beneficial during infection, NETs can unfortunately have harmful consequences for the organism and thus participate in the pathogenesis of several human diseases. These include autoimmune and inflammatory diseases such as systemic lupus erythematous (SLE), rheumatoid polyarthritis and cardiovascular diseases such as acute stroke or myocardial infarction.

## 4. Models of Thrombosis

### 4.1. Animal Models

Animal models are used to study the mechanisms of thrombosis. Many models have been developed and vary according to the species and vascular area targeted. This paragraph summarizes these different models.

As shown in Table 1, the main species used to study thrombosis are zebrafish [25,26,27], mice [8,28,29,30,31,32,33], rats [34,35], rabbit [36,37,38] hamsters [39,40], guinea pigs [41,42], pigs [43,44,45], dogs [46,47,48], and baboons [9,49,50]. Of course, the mouse remains the reference animal model in the study of cardiovascular disease for several reasons: (1) it is a mammalian species with many physiological similarities to humans, (2) it is economical to house, (3) it is easy to manipulate, and (4) its genome is easily modifiable, allowing the creation of numerous transgenic lines [25].

Severe human diseases have pushed us to extend the scope of scientific study, which is why many arterial and venous thrombosis models have been developed. In Table 1, you can see the many vascular areas studied in animal models. Venous thrombosis is a biological phenomenon that is widely studied by scientists. Many pathologies are associated with it, which is why it has been studied in many vascular areas. Among the best characterized veins, we found the inferior vena cava [8,9,43], jugular vein [38,46], and femoral vein [8,35,41]. For arterial thrombosis, the most studied vessel is the carotid artery [29,32], although the mesenteric [28,31] and cremasteric [28] arterioles are sometimes used.

### 4.2. Experimental In Vivo Thrombosis Models

Animal models of thrombosis are valuable tools in understanding the cellular and molecular mechanisms involved in the pathogenesis of thrombus formation. Many methods are commonly used to experimentally initiate thrombosis using in vivo animal models. The associated mechanisms are varied and complex depending on the injury induced. Photochemical thrombosis with rose bengal and ferric chloride (FeCl_3_) injury are known and reputed models to induce thrombosis after denudation of the endothelium. This step is notably present in the biological process of atherosclerotic plaque rupture involved/present in several human diseases. Other models, such as the laser thrombosis model and the deep vein thrombosis (DVT) model, maintain the integrity of the vascular endothelium and activate it. There are also so-called “spontaneous” thrombosis models. These models consist of the use of animals genetically modified for the proteins involved in coagulation. In particular, we found in the literature mouse lines with modifications of the sequences coding for tissue factor pathway inhibitor (TFPI), antithrombin (AT), protein C (PC), and its entire signaling pathway, including thrombomodulin (TM), endothelial protein C receptor (EPCR), Factor XIII [51] and Factor V (or Factor V Leiden, FVL) [52].

Together, these models give us a better overview and a broad and rich repertoire of knowledge about the phenomenon of thrombosis. The models listed below are among the most well-known and mastered in the scientific world, putting forward a mechanism dependent on neutrophils and/or NETs.

## 5. Models of Thrombosis by Endothelium Denudation

Numerous models are described in the literature as inducing thrombosis by a mechanism of denudation of the endothelium. These are notably found in several human pathologies, such as atherosclerotic plaque ruptures and aneurysm ruptures. A better understanding of this frequent biological/pathological phenomenon is crucial to improve future therapeutic targets, and animal models are precious tools in this field. Models inducing thrombosis by denudation of the endothelium are studied below:

### 5.1. The Ferric Chloride (FeCl_3_) Chemical Thrombosis Model

**Figure 1:**
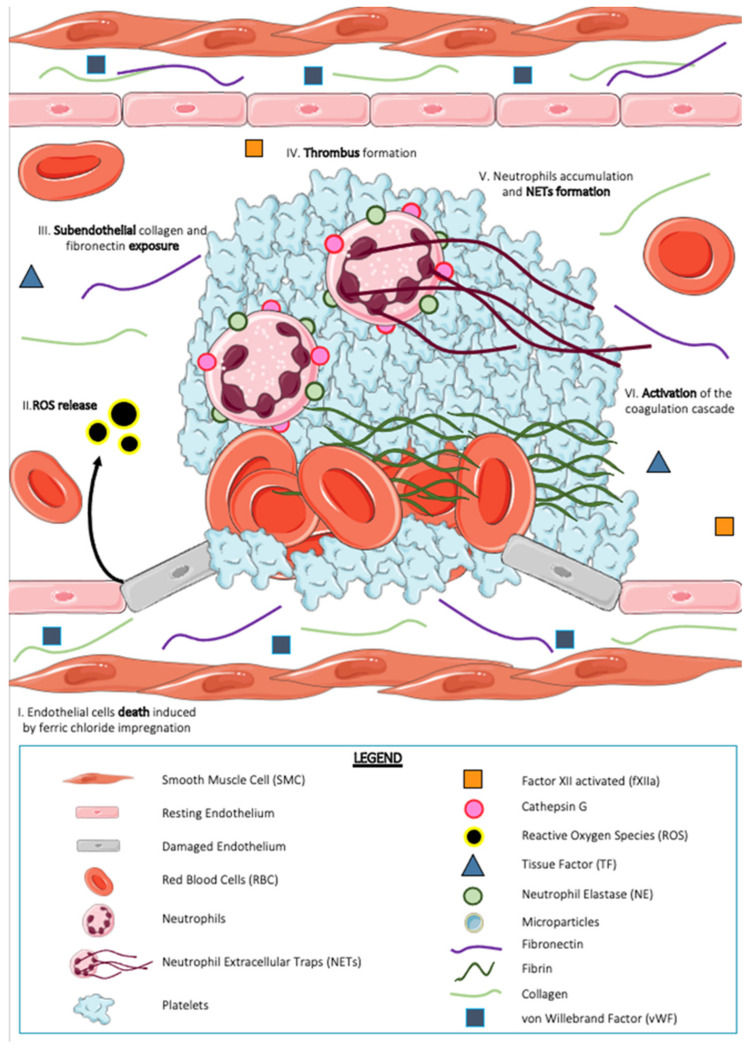
Thrombus formation after ferric chloride (FeCl_3)_ exposure. In the FeCl_3_ exposure model, the blood vessel wall is exposed to iron chloride for a few minutes, usually less than ten minutes. This exposure causes cell death in the vascular endothelium (I) and generation of reactive oxygen species (ROS) (II). All the blood component and cells are exposed to the subendothelial matrix (III) (collagen, fibronectin, von Willebrand factor). This leads to platelet activation via an GPVI-collagen interaction and thus to their aggregation; some red blood cells could be trapped in the thrombus (IV). Neutrophils accumulate in the growing thrombus and form neutrophil extracellular traps (NETs) (V). The activation of the coagulation cascade is tissue factor (TF)-dependent in this model (VI) and leads to fibrin generation.

The FeCl_3_ thrombosis model (Figure 1) is a chemical thrombosis model that consists of the impregnation of FeCl_3_ on the vessel of interest. FeCl_3_ is initially soaked onto Whatman paper, which is then deposited on the area of interest of thrombosis. Thrombus formation in this model is dependent on the release of ROS from the secreted endothelium following significant oxidative stress that can induce endothelial cell death and exposure of subendothelial collagen [53]. The FeCl_3_ thrombosis model is a well-known model but is criticized by scientists because of the large number of parameters to be defined [54]. Indeed, this model can be performed on microvessels [28] (arterioles and venules of the mesentery, cremaster) and on macrovessels [55,56] (the carotid artery). Additionally, a few labs reported the use of a skinfold chamber to study thrombosis and thrombolysis following a FeCl_3_ or a photochemical injury [32,57]. The FeCl_3_ molecule to be chosen (anhydrous or aqueous), as well as its concentration (from 2.5 to 50%, the most common being 10%), can vary the results thus obtained following this surgery. Finally, the time of application (from one to five min) on the vessel of interest is a parameter to be studied. The frequently used animal models are rats, mice, and rabbits. All of these criteria and parameters make the FeCl_3_ thrombosis model difficult to standardize. Indeed, this model presents controversial mechanics with either denudation of the endothelium [28,58] or conservation of the integrity of endothelial cells [55,56]. In the case where injury induces denudation, the model would involve numerous pro-coagulant molecules contained in the subendothelial matrix, such as collagen, its receptor GPVI, and fibronectin, thus leading to the formation of an occlusive thrombus. Authors have revealed a positive correlation between the concentration of FeCl_3_ and the size of the thrombus. The higher the concentration, the larger and more occlusive the thrombus. In the case where injury does not induce exposure of the subendothelial matrix, the FeCl_3_ model is shown to be dependent on von Willebrand factor (VWF) [56].

Indeed, the use of VWF-deficient mice resulted in a 50% reduction in thrombus size in the carotid artery following FeCl_3_ impregnation [56]. Regarding the activation of the coagulation cascade and the generation of fibrin, this chemical model of thrombosis is known to be dependent on two pathways: extrinsic involving tissue factor [56] and intrinsic with fXII [49]. Interestingly, this model highlights the involvement of red blood cells in thrombosis. Indeed, erythrocytes, which are rarely described in the world of thrombosis, are used in this model as a mediator of platelet adhesion to the endothelium [55]. Other cellular components, such as platelets, have been studied. It appears that a platelet activation gradient exists in thrombi formed by FeCl_3_, dividing the platelets into two subpopulations: an activated population, known as procoagulant and degranulated, and a nonactivated population containing granules [59]. In this model, neutrophils have also been described as involved in thrombus formation and in the activation of the coagulation cascade in vivo.

In 2010, Massberg et al. used a carotid artery FeCl_3_ model in mice deficient in neutrophil elastase (NE) and cathepsin G (named *Elane*−/−; *Ctsg*−/−) compared to wild-type (WT) control mice to show that the activation of the coagulation cascade is altered in mice deficient in these two serine proteases from neutrophils [12]. Indeed, some parameters, such as thrombus size (−75%), bleeding time (−65%), and fibrin generation (−60%), were significantly reduced in *Elane*−/−; *Ctsg*−/− *mice* compared to control mice. A partial KO mouse model, i.e., NE deficient but with normal cathepsin G expression, revealed a decrease in fibrin generation similar to *Elane*−/−; *Ctsg*−/− *mice*, thus suggesting that intravascular fibrin formation is predominantly related to NE. In 2013, Faraday et al. showed the involvement of neutrophil-derived cathepsin G in platelet thrombus formation following FeCl_3_ injury to the mouse mesenteric arterial microcirculation [60]. After indirectly showing in vitro in human PRP that cathepsin G promoted platelet aggregation by using an inhibitor (cathepsin G inhibitor I), the authors continued their in vivo study in mice. Injection of cathepsin G inhibitor I or the use of cathepsin G-deficient mice decreases the capacity of platelets to accumulate at the site of injury and therefore significantly increases the time of thrombus formation (occlusion time twice as long compared to control mice). Leal et al. in 2017 also discussed the involvement of NETs in a FeCl_3_ model associated with an orthotopic mouse breast cancer model [61]. Indeed, immunohistochemical sections of induced thrombi in carotid arteries revealed the presence of Ly6G+ cells colocalizing with extracellular DNA labeling, strongly suggesting the involvement of NETs in this model of thrombosis.

### 5.2. The Rose Bengal Photochemical Thrombosis Model

**Figure 2:**
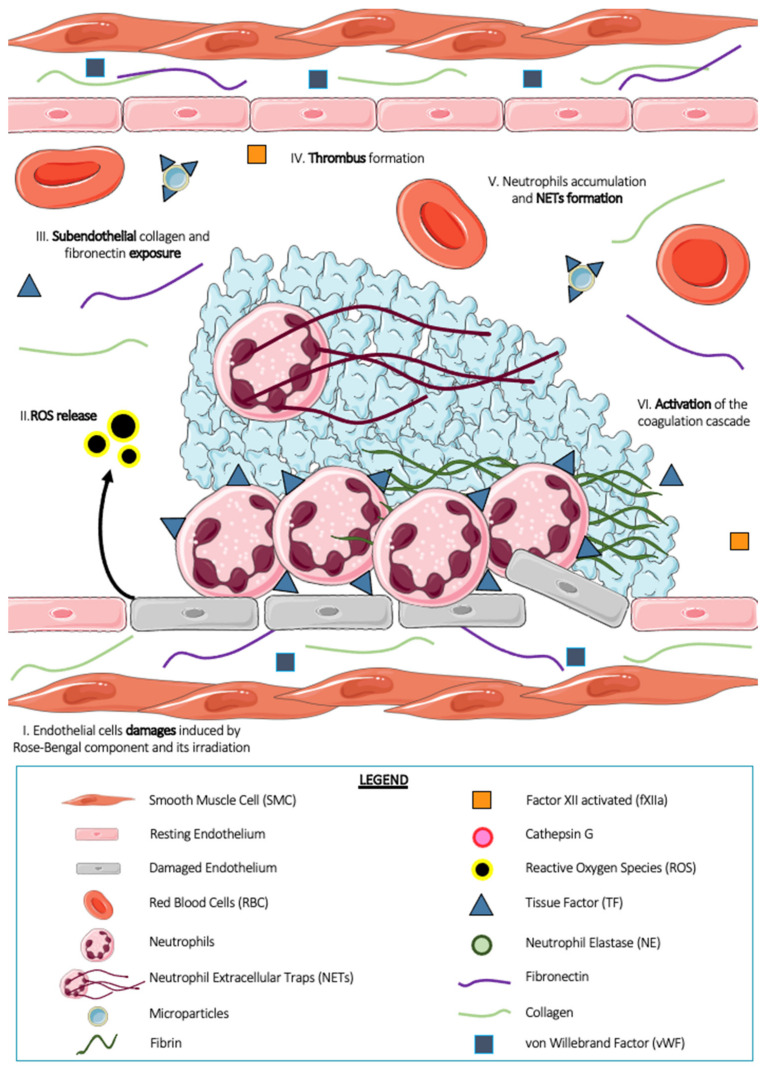
Thrombus formation after rose bengal photosensitization. The vessel wall is exposed to photosensitized rose bengal-induced phototoxicity, that leads to damage of the vascular endothelial cells (I) inducing permeability, loss of protein expression, release of reactive oxygen species (ROS) (II), which may even lead to cell death. This results in partial or complete denudation of the endothelium accompanied by the exposure of subendothelial collagen and fibronectin (III). These strongly pro-thrombotic compounds induce the formation of a thrombus (IV) in which leukocytes are identifiable (V). These leukocytes, like neutrophils, carry on their surface tissue factor (TF) responsible for the generation of fibrin in this model (VI).

The rose bengal thrombosis model (Figure 2) is a two-step thrombosis model. It consists of intravenous injection into the systemic circulation of the photosensitive chemical compound rose bengal, followed by irradiation of the area of interest with a laser at 543 nm wavelength. Photoactivation of the rose bengal compound by the laser results in the generation of ROS, which induces peroxidative chemical damage in the endothelial membrane [62]. These ROS are the initial stimuli involved in platelet activation and thrombus formation in this model [63]. Thrombus formation in this model is followed from a few hours to several days, with a plateau reached after Day 21. Similar to chemical thrombosis with FeCl_3_, photochemical thrombosis with rose bengal is known but little used in the scientific community because of its reproducibility problems. Indeed, the concentration of rose bengal used (from 10 to 50 mg/kg, the most common being 50 mg/kg), the type of injection (bolus and/or infusion), the choice of laser and its duration (between 20 to 40 sec or even continuous) and its intensity are among the many parameters to be established before performing the experiments [54]. This model can be applied to several vascular beds, notably the carotid artery [64], femoral artery [65], and arterioles of the mesentery [63], and can be performed in several animal models, such as zebrafish [66], guinea pigs, and mice [27,62,63]. Perez et al. showed, in 2014, that thrombus size and percentage of occlusion were related to the concentration of rose bengal administered to the mouse [63]. Indeed, by studying four different concentrations (5, 10, 25, and 50 mg/kg), the authors highlighted thrombotic events from the administration of 25 mg/kg rose bengal and a reduced occlusion time to 30 min for the concentration at 50 mg/kg. Thrombus formation in this model is dependent on integrin α2β1 and the collagen receptor GPVI [67]. In 2008, a team of researchers concluded that platelets were not required for cerebral infarct formation in mice following rose bengal photothrombosis [68]. Indeed, using antibodies blocking platelet aggregation functions (JON/A) or antibodies allowing platelet depletion (GPIb), the authors revealed no changes in infarct size or in the kinetics of infarct development compared with control mice. In addition, experiments using FXII-deficient mice and FXa blocking molecules (a low molecular weight heparin, fraxiparin) revealed that activation of the coagulation cascade was independent of the intrinsic pathway in this model. In addition, another team using TF−/− mice revealed that the main source of TF in this model is derived from vascular endothelial cells and not from leukocytes or leukocyte microparticles, as shown by many other teams [69].

Among the cellular actors studied in this photochemical model, neutrophils and particularly NETs are strongly involved in platelet thrombus formation. In 2005, Shimazawa et al. showed an accumulation of leukocytes and, more particularly, neutrophils on the injured endothelium following photochemical thrombosis in rose bengal [70]. Moreover, from three days after induction of injury, these neutrophils, in addition to being adhered to the extracellular matrix, interact with platelets, suggesting a potential role in thrombus formation in vivo.

Regarding NETs, in 2017 Monteiro’s team showed the involvement of NETs in the rose bengal model of thrombosis to better characterize the cellular phenomena of cancer-associated thrombosis [61]. The authors demonstrated in an orthotopic mouse breast cancer model that treatment with Deoxyribonuclease I (DNAse I) prior to the induction of photochemical thrombosis significantly reduced the occlusion time compared to control mice also treated with DNAse I. This suggests the involvement of NETs in cancer-related thrombosis in this model.

## 6. Models of Thrombosis by Endothelium Activation

In addition to thrombosis models generated by denudation of the endothelium, innovative techniques have made it possible to develop new thrombosis models by activating endothelial cells. These new models allow the study of a larger number of human pathologies in which the mechanisms are not yet elucidated, such as phlebitis of the lower limbs and thrombosis associated with cancer. The models commonly known to induce thrombosis by activation of endothelial cells are listed below.

### 6.1. The Ablative Laser Thrombosis Model

**Figure 3:**
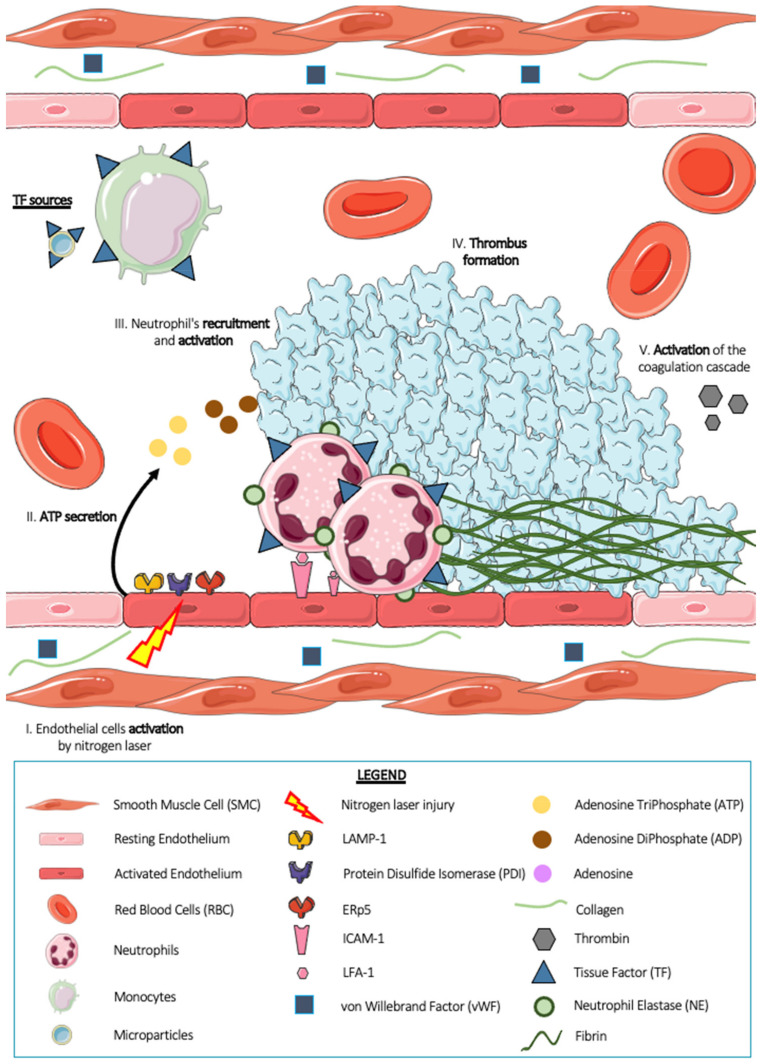
Thrombus formation after a nitrogen laser-induced injury. Platelets, red blood cells, neutrophils, monocytes, microparticles as well as plasma proteins and coagulation factors circulate into the bloodstream. When the endothelium cells are activated by a nitrogen laser beam (I), they secrete adenosine triphosphate (ATP) (II). ATP is a known agonist of neutrophils. It allows their activation and promotes their adhesion to the endothelium (III). Activated neutrophils carry on tissue factor (TF) on their surface responsible for activation and aggregation of platelets. Moreover, platelets secreted adenosine diphosphate (ADP) during their activation, which promotes their aggregation and their accumulation at the site of injury and thus leads to the formation of thrombus (IV). Thrombin, initially present at the site of injury in small quantities which increases with the secretion of active platelets, will induce, together with ATP, ADP, and tissue factor molecules, the activation of the coagulation cascade and the generation of fibrin (V).

The ablative laser thrombosis model (Figure 3) was developed by Bruce and Barbara Furie [71]. It is a well-known model, but is considered delicate by scientists [54]. It consists of a 440 nm nitrogen laser injury (170 J shot) on the arterial endothelium of the mouse cremaster microcirculation. Classically, one to two pulses are necessary to form a thrombus. The advantages of this thrombosis model are numerous: (1) the ability to monitor thrombus formation in time and space, (2) the ability to generate multiple thrombi in the same animal, (3) the ability to follow the kinetics of thrombus formation in vivo, and (4) the ability to quantify several thrombosis parameters simultaneously [72]. The use of a calcium mobilization reporter compound, Fluo-4-AM, demonstrates that laser injury induces rapid activation of targeted endothelial cells [73]. This activation also generates degranulation of the endothelium expressing the molecules Lysosomal-Associated membrane protein-1 (LAMP-1) [73], PDI [74], and ERp5 [75] on its surface. The endothelium is a key player in this model of thrombosis, with the exception of the subendothelial matrix and its pro-thrombotic components. Indeed, Dubois et al. demonstrated in 2006 and 2007 that the mechanics associated with the laser thrombosis model are independent of the subendothelial matrix (collagen, VWF), notably by using GPVI deficient mice [28] and *VWF*−/−*KO mice* [76]. This model usually induces small or even intermediate but nonocclusive thrombi. Thrombus formation is a rapid process in this model: the thrombus peaks in size approximately 2 min after injury and then decreases until it disappears completely [28,76].

Further studies on the activation of the coagulation cascade and the generation of fibrin have revealed that circulating tissue factor is involved, or, more precisely, tissue factor carried by microparticles [77,78]. Platelets are slightly more reserved in this model. Indeed, two studies have shown a rather passive and inhibitory involvement of platelets in fibrin generation. Vandendries et al., using protease-activated receptor 4-deficient mice (PAR4−/−), i.e., mice with a deficiency in the thrombin receptor on their platelets, revealed unchanged fibrin generation compared with control individuals [79]. Falati et al. demonstrated that the adhesion molecule Platelets Endothelial Cell Adhesion Molecule-1 (PECAM-1) present on the surface of platelets would have an inhibitory effect on thrombus formation [30]. In addition to the main effectors known in the world of thrombosis, such as platelets and the endothelium, neutrophils have been described as strongly involved in this model of laser thrombosis.

**Figure 4:**
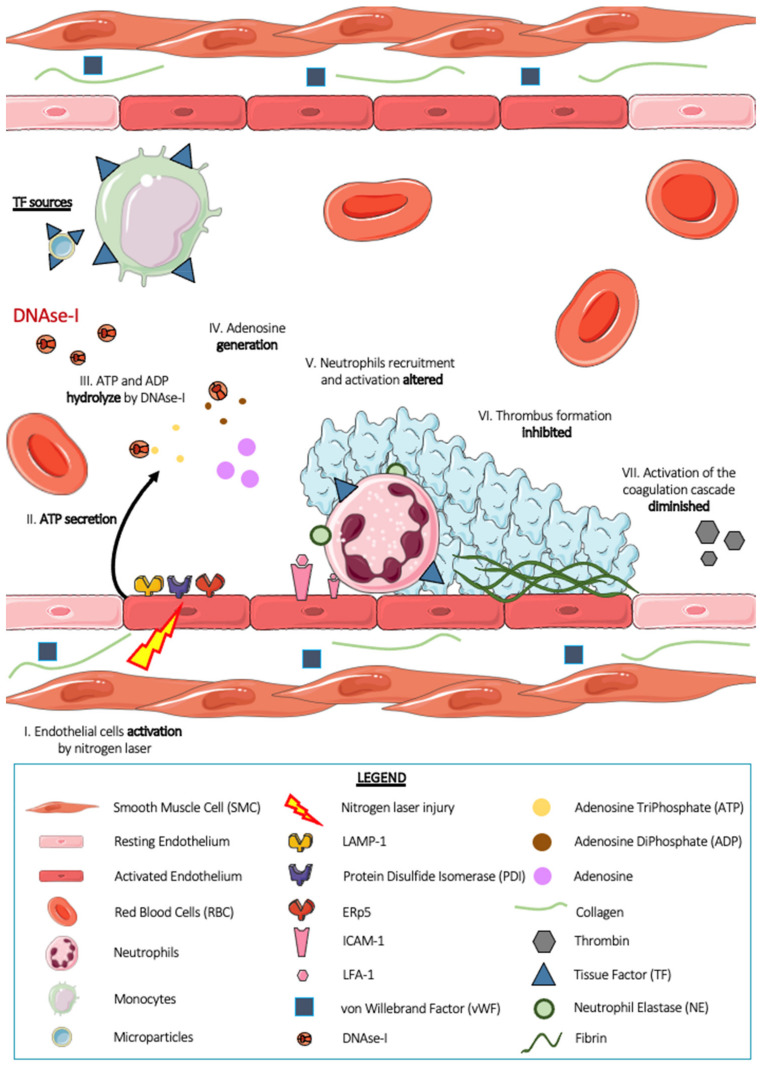
Thrombus formation after a nitrogen laser induced injury in presence of deoxyribonuclease (DNAse-I). When the endothelium is activated by a nitrogen laser blast (I), it releases adenosine triphosphate (ATP) (II). ATP and adenosine diphosphate (ADP), two known agonists of neutrophils and platelets, are both degraded by DNAse I injected into the general circulation of mice (III). This hydrolysis induces the generation of adenosine (IV), an antagonist of neutrophil activation. Neutrophil recruitment and activation are impaired (V). Thrombus formation is then inhibited (VI). Activation of the neutrophil-dependent coagulation cascade and thus the formation of fibrin is strongly reduced after injection of DNAse I (V).

Darbousset et al. in 2012 showed that neutrophils are the first cells present at the injury site, arriving before platelets [7]. The neutrophil–endothelial cell interaction is therefore the first step in arterial thrombus formation in this model. Intravital microscopy experiments in mice revealed that this interaction is dependent on the IntraCellular Adhesion Molecule-1 (ICAM-1)/Lymphocyte Function Associated antigen-1 (LFA-1) ligand–receptor system and that depletion of platelets from animals by intravenous injection of R300 did not affect neutrophil accumulation. In addition, the use of a calcium mobilization reporter compound, Fluo-4-AM, revealed that neutrophils accumulated at the site of endothelial injury were activated. They release granular contents such as serine protease NE [24] by exocytosis and express tissue factor on their surface either by de novo production or by acquisition via monocytes or TF+ microparticles. The procoagulant TF+ activity of these neutrophils is still under debate. However, when neutrophil–endothelial cell interaction is no longer allowed using an ICAM-1 blocking antibody, tissue factor accumulation is strongly decreased, suggesting that neutrophils would be the main source of TF in the laser thrombosis model. The authors also showed the importance of adenosine triphosphate (ATP), which plays an indispensable role in the recruitment and activation of neutrophils in the vascular endothelium, and the activated endothelium is a potential physiological source of ATP [2]. Finally, in 2021, our team demonstrated the involvement of NETs in this laser thrombosis model [24]. Indeed, intravital microscopy experiments showed that the injection of DNAse I (Figure 4), as well as the injection of apyrase, a known ATPase, into the general circulation of WT mice induced a strong decrease in the size of the thrombus after laser beam injury, thus suggesting a possible involvement of NETs in this model. Thrombi formed in WT mice were analyzed by electron microscopy, and the images revealed the presence of platelets and neutrophils within the thrombus. No NETs were formed. In vitro experiments demonstrated that DNAse I has the ability to hydrolyze molecules with phosphodiester bonds present in DNA but also in molecules such as adenosine triphosphate (ATP) and adenosine diphosphate (ADP), which are two known agonists of neutrophils and platelets. In this article, we also underlined the difficulty of demonstrating the presence of NETs without specific markers. Indeed, the markers classically used in the scientific community seem to be markers of activated neutrophils (CitH3, NE) rather than NETs. This is a real problem and can sometimes lead to premature and unreliable conclusions.

### 6.2. The Vena Cava Ligation Model (DVT)

**Figure 5:**
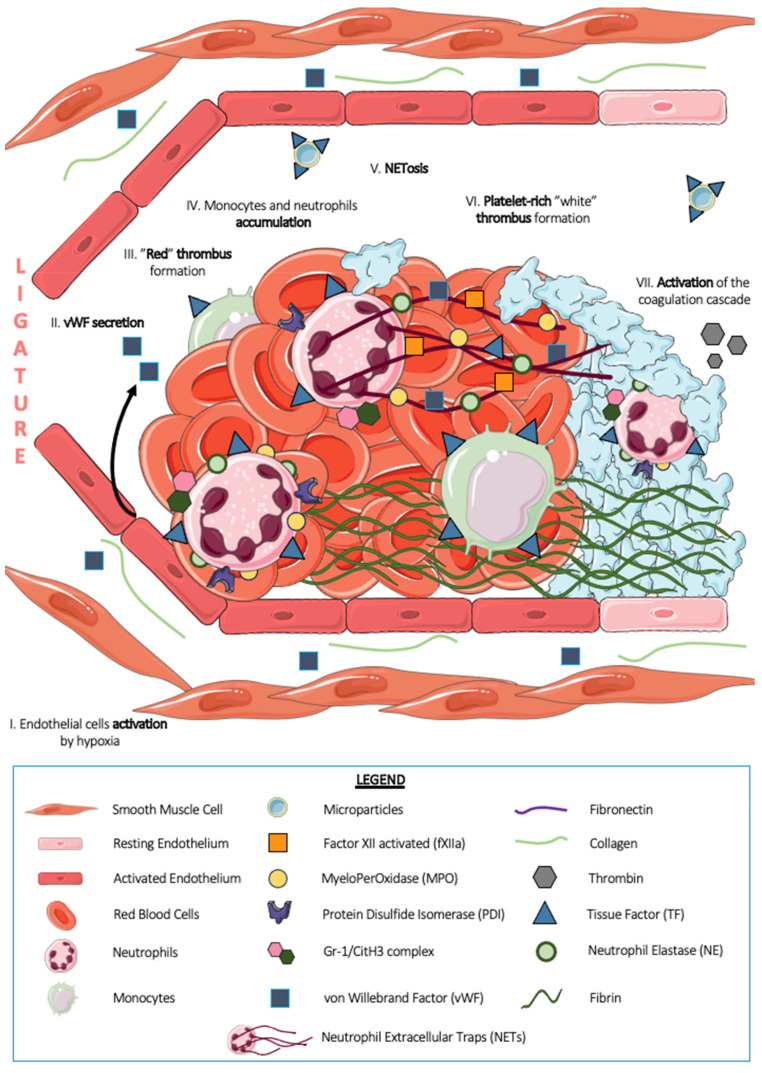
Thrombus formation after a ligature of the inferior vena cava (DVT model). Following a 90% flow restriction with a ligature of the inferior vena cava (IVC) in mice, the induced stenosis causes turbulences in blood flow and hypoxia of the cells, which together lead to local activation of the endothelium (I). This activation induces the release of von Willebrand factor initially contained in the Weibel–Palade bodies of endothelial cells (II). This pattern leads initially to the formation of a so-called “red” thrombus rich in erythrocytes (III). In this part of the thrombus, immune cells such as neutrophils and monocytes, as a majority, accumulate (IV).

The deep vein thrombosis (DVT, Figure 5) model was mainly developed by Professor Denisa Wagner in the United States (Boston). Classically, this model consists of partial (stenosis) or total (stasis) ligation of the inferior vena cava (IVC) in mice [80] for a few hours (one to 48 h) to a few days (one to 16 days) [81]. The turbulence of blood flow due to ligation leads to endothelial dysfunction. Indeed, the stasis induced in the vicinity of the ligation leads to hypoxia, which in turn contributes to the local activation of endothelial cells and venous thrombogenesis [3]. The composition of the thrombus thus formed has been well described in the literature, with a “red” thrombus rich in erythrocytes located proximal to the ligation and a “white” thrombus rich in platelets located more distal to the ligation [80]. Circulating cells involved in thrombus formation, such as platelets, leukocytes, and red blood cells, interact with an intact endothelium: denudation of the endothelium and exposure of the subendothelial matrix have never been described in this model, thus conferring a collagen-independent but tissue factor-dependent mechanism [8]. Furthermore, it appears that platelet thrombus formation in this model is dependent on von Willebrand factor secreted by Weibel–Palade bodies of the endothelium locally activated by ligation [80]. In addition, another study showed a positive regulation of venous thrombogenicity by P-selectin and a negative regulation of leukocyte-derived microparticles [82]. Using animal models, the authors determined that the overexpression of P-selectin was associated with a high risk of venous thrombosis and that leukocyte microparticles amplified the phenomenon of venous thrombosis by their expression of tissue factor. Surprisingly, a recent article by DeRoo et al. demonstrated that platelets are not essential for thrombus formation in the DVT model [81]. Indeed, platelet depletion induced by the injection of R300 (a cocktail of antibodies containing an anti-GPIb) in mice with inferior vena cava ligation does not generate a decrease in either the size or the volume of the thrombus thus formed. Among the components of these thrombi, Fuchs et al. described that extracellular DNA was present within the thrombi formed by DVT, which was revealed from immunohistochemistry sections [10]. The study of the composition of these thrombi is still ongoing and aimed at the development of new therapeutic strategies to combat deep vein thrombosis in humans. In 2012, Von Bruhl et al. studied the involvement of monocytes, platelets, and neutrophils in platelet thrombus formation in this model [8]. Indeed, the authors demonstrated that leukocytes were the majority cell population present in thrombi from inferior vena cava ligation for 48 h. More than 70% of these leukocytes expressed a double Ly6G+ MPO+ label, suggesting the presence of neutrophils. The total depletion of neutrophils by injection of the Ly6G antibody at high concentrations inhibits experimental thrombosis by ligation of the inferior vena cava, thus suggesting the importance of this cellular actor in thrombus formation. By using genetically modified *LysM-eGFP* mice with Ly6G labeling, the authors showed that neutrophils represented more than 80% of the cells recruited six hours after ligation, i.e., in the early phase of venous thrombus formation. Monocytes, Ly6G- F4/80+ cells, represent the remaining leukocyte population. In the same year, Brill et al., using staining on frozen thrombi sections, localized activated neutrophils in the “red” part of the thrombus by a double-positive Gr-1/CitH3 marker, thus making activated neutrophils the pioneer cell initiating thrombosis in the DVT model [9]. The use of a mouse model depleted in TF in neutrophils and monocytes allowed the authors to show that these two leukocyte populations express tissue factor required for the development of venous thrombosis in this model. In 2013, Martinod et al. described that a modification of neutrophil histones was essential for neutrophil activation and amplification of the thrombotic phenomenon associated with DVT [83]. Indeed, *PADI4*−/− *mice*, which are deficient in the enzyme peptidylarginine deiminase 4 (PAD4), responsible for chromatin decondensation and histone citrullination, are protected from thrombotic events in large veins such as the inferior vena cava. These experiments make activated neutrophils essential for platelet thrombi formation in the DVT model.

Finally, neutrophils fuel venous thrombogenesis by releasing NETs. The article by Professor Denis Wagner showed in 2012 that in vivo, after three hours of blood flow restriction by ligation of the inferior vena cava, immunofluorescence of thrombi revealed extracellular DNA close to Ly6G+ MPO+ NE+ cells, thus suggesting that this DNA belongs to neutrophils. Interestingly, these markers disappear upon neutrophil depletion in mice, indicating that neutrophils are the major source of NETs during the development of DVT. Similar to neutrophils, NETs are decorated with TF and PDI, making this structure a prothrombotic surface. Confocal microscopy experiments revealed that NETs were able to bind FXII to their surface and to serve, thanks to the negative charges provided by the DNA, as a carrier for its activation (FXIIa). Venous thrombus formation is indeed significantly reduced in FXII-deficient mice and mice treated with a FXII-inhibiting compound, thus demonstrating the FXII-dependent profile of this model [8]. In 2010, Fuchs et al. used a baboon iliac vein DVT model to show that NETs support platelet adhesion and fibrin generation in a thrombin-dependent manner. Indeed, NETs are able to bind plasma proteins, such as VWF, fibronectin, and fibrinogen, which are important for thrombus stability [10]. Very interestingly, in 2013, Martinod et al. showed that NET formation in the DVT model was PAD4-dependent but NE-independent [84]. Indeed, using NE-deficient mice, the authors still revealed the presence of NETs in thrombi formed following inferior vena cava ligation in these mice. The mechanism associated with NETosis in thrombosis remains to be clarified. What has been reported in the literature is that NETosis in the DVT model is dependent on soluble P-selectin found in plasma or that contributed by platelets. To define NETs as an essential component of DVT, the use of DNAse I, an enzyme known to hydrolyze phosphodiester bonds contained in DNA, is often applied. In their 2012 paper [8], Professor Wagner’s team administered DNAse I to mice undergoing partial inferior vena cava ligation surgery. After 48 h, smaller, lighter thrombi were revealed, with disappearance of double-positive filamentous structures for DNA and neutrophil markers. These experiments thus underscored the importance of NETs in deep venous thrombosis.

## 7. Clinical Relevance

Since the discovery of NETs, their detection has posed a real challenge. As previously mentioned, the classical markers used are the citrullination of histone H3 by the enzyme PAD4, the overexpression of NE and MPO, and DNA secretion in the extracellular compartment. However, these markers are not specific for NETs and may also indicate the presence of activated neutrophils. In vivo, another method such as DNAse I infusion is used to show the involvement of NETs: this allows the study of the disease progression after injection and to record the effects of NETs and their removal in the animal. This was first described in the pathology of sepsis [85]. Like neutrophils, NETs have a double identity: they are beneficial for the organism by limiting the dissemination and by causing the death of bacterial pathogens; but they are also detrimental because they cause numerous damages to organs (lungs, liver, kidneys…) and vessels [86]. Recently, Prof. Chen’s team demonstrated that in a mouse model of airway inflammation with mucus hypersecretion after an injection of LPS, the NET structures present in the murine lungs tended to disappear after treatment with DNAse I, improving the condition of the animals too [87]. Still in animal models, the benefit of this treatment has also been demonstrated in Transfusion-Related Acute Lung Injury (TRALI) [88], diffuse alveolar hemorrhage (a complication of systemic lupus erythematous) [89], thrombosis [9], and thrombosis associated with cancers. However, we recently demonstrated that DNAse I could act as an ATPase independent of NETosis [24]. In 2014, Ho and colleagues [90] showed that sivelestat, a molecule acting on the early stages of NETosis through the inhibition of NE, led to a significant decrease in tumor growth and progression (tumor volume reduced by nearly 40% in mice).

Clinically, NETs are also extensively studied. Unfortunately, they are most often associated with poor prognosis and increased patient mortality [91]. Therefore, more and more tests and assays are being developed to measure NETs. It is important to emphasize that this involves the assay of NET-derived products, such as extracellular DNA, extracellular DNA and its complex with MPO, CitH3, and NE in the blood of patients. Among the different pathologies where these markers have been tested, cancers (breast, lung, and colorectal) [92], stroke [93], deep vein thrombosis [94], and myocardial infarction [95] are listed. In these publications, patients have increased plasma levels of NE, CitH3, circulating DNA, and MPO compared with healthy volunteers. More recently, the elevation of these markers has similarly been demonstrated in the plasma of patients with COVID-19 [96]. Concerning therapeutics, only cardiorespiratory pathologies, primarily cystic fibrosis, requires limiting the effects of NETs on the organism. Indeed, since the 1090s, DNAse I (Pulmozyme) has been administered by air to patients suffering from poor pulmonary condition [97]. This molecule improves pulmonary functions, depolymerizes extracellular DNA, and reduces the viscoelasticity of air secretions [98]. Several studies subsequently mentioned that the extracellular DNA present in the lungs of these patients would be of neutrophilic origin, thus indicating NETs as deleterious participants in this pathology. Conversely, for cardiovascular diseases and cancers, treatments consisting of antiplatelet or anticoagulant drugs (clopidogrel, aspirin, heparin) associated with chemotherapy (with or without surgery) remain the best therapy to date.

## 8. Conclusions

This review covers the different mice models of thrombosis used by the scientific community revealing the involvement of neutrophils and NETs in thrombosis. Preclinical mouse models are the closest models to humans that can be used in research. However, some differences may exist between human and murine proteins, so in rare cases the direct translation of preclinical results to the human clinical studies may be disappointing. Therefore, there are numerous mouse preclinical models with various mechanisms: some induce denudation of the endothelium, while others induce its activation, and this occurs in two vascular domains: arteries and veins. This study reveals that for more than a decade, neutrophils, in addition to their immune activity, have been shown to play an important role in thrombosis, giving rise to the phenomenon of immunothrombosis. Indeed, neutrophils express TF on their surface, a pioneer molecule of the extrinsic activation pathway of the coagulation cascade. Moreover, the serine proteases contained in the neutrophil granules released during its activation, such as NE, MPO, or cathepsin G, are enzymes capable of hydrolyzing TFPI, an inhibitor protein of TF, once again promoting the activation of the coagulation cascade. Finally, neutrophils express the PDI protein on their surface, promoting the activation of tissue factor located on the surface of neutrophils, thus conferring triple coagulant activity on neutrophils.

Similar to neutrophils, NETs seem to be involved in thrombus formation. Indeed, the neutrophil DNA released into the extracellular compartment following its explosion would serve as a support for platelet aggregation and fibrin generation. This highly adhesive surface would trap proteins contained in the plasma, such as fibronectin and fibrinogen, thus ensuring the stability of the thrombus.

Together, neutrophils and NETs are strongly involved in thrombosis. The study of the composition of thrombi allows the design of new targeted and innovative therapeutic strategies to reduce mortality due to these thrombotic events.

## Figures and Tables

**Table 1:** Summary of species and vascular beds used in thrombosis models.

Specie	Vessel	Reference
Zebrafish	Caudal vein, dorsal aorta	Jagadeeswaran et al. [25] Fish et al. [26] Lee et al. [27]
Mouse	Carotid artery, vena cava, jugular vein, femoral artery, cremasteric arterioles, mesenteric arterioles, dorsal arterioles and venules	Dubois et al. [28] von Brulh et al. [8] Denis et al. [29] Falati et al. [30] Mangin et al. [31] Wang et al. [32] Boulaftali Y [33]
Rat	Carotid artery, femoral artery	Nayak et al. [34] Bang et al. [35]
Rabbit	Carotid artery, aorta, jugular vein	Liu et al. [36] Costa et al. [37] Shinozawa et al. [38]
Hamster	Carotid artery, femoral artery	Jankowski et al. [39] Nemmar et al. [40]
Guinea pig	Femoral artery, cerebral artery	Takiguchi et al. [41] Moriguchi et al. [42]
Pig	Vena cava, mesenteric arterioles, coronary artery	Hosaka et al. [43] Kai et al. [44] Dogne et al. [45]
Dog	Jugular vein, femoral artery, coronary artery	Frisbie et al. [46] Badylak et al. [47] Bjorkman et al. [48]
Baboon	Vena cava, femoral artery	Brill et al. [9] Matafonov et al. [49] Young et al. [50]

## References

[1] Darbousset R., Mezouar S., Dignat-George F., Panicot-Dubois L., Dubois C. (2014). Involvement of Neutrophils in Thrombus Formation in Living Mice. Pathol. Biol..

[2] Darbousset R., Delierneux C., Mezouar S., Hego A., Lecut C., Guillaumat I., Riederer M.A., Evans R.J., Dignat-George F., Panicot-Dubois L. (2014). P2X1 Expressed on Polymorphonuclear Neutrophils and Platelets Is Required for Thrombosis in Mice. Blood.

[3] Fuchs T.A., Brill A., Wagner D.D. (2012). NET Impact on Deep Vein Thrombosis. Arterioscler. Thromb. Vasc. Biol..

[4] Kubes P. (2018). The Enigmatic Neutrophil: What We Do Not Know. Cell Tissue Res..

[5] Liew P.X., Kubes P. (2019). The Neutrophil’s Role During Health and Disease. Physiol. Rev..

[6] Brinkmann V. (2004). Neutrophil Extracellular Traps Kill Bacteria. Science.

[7] Darbousset R., Thomas G.M., Mezouar S., Frère C., Bonier R., Mackman N., Renné T., Dignat-George F., Dubois C., Panicot-Dubois L. (2012). Tissue Factor–Positive Neutrophils Bind to Injured Endothelial Wall and Initiate Thrombus Formation. Blood.

[8] Von Brühl M.-L., Stark K., Steinhart A., Chandraratne S., Konrad I., Lorenz M., Khandoga A., Tirniceriu A., Coletti R., Köllnberger M. (2012). Monocytes, Neutrophils, and Platelets Cooperate to Initiate and Propagate Venous Thrombosis in Mice In Vivo. J. Exp. Med..

[9] Brill A., Fuchs T.A., Savchenko A.S., Thomas G.M., Martinod K., De Meyer S.F., Bhandari A.A., Wagner D.D. (2012). Neutrophil Extracellular Traps Promote Deep Vein Thrombosis in Mice: NETs Promote Deep Vein Thrombosis. J. Thromb. Haemost..

[10] Fuchs T.A., Brill A., Duerschmied D., Schatzberg D., Monestier M., Myers D.D., Wrobleski S.K., Wakefield T.W., Hartwig J.H., Wagner D.D. (2010). Extracellular DNA Traps Promote Thrombosis. Proc. Natl. Acad. Sci. USA.

[11] Bassuk J.A., Capodici C., Berg R.A. (1990). Protein Disulphide Isomerase from Human Peripheral Blood Neutrophils. J. Cell. Physiol..

[12] Massberg S., Grahl L., von Bruehl M.-L., Manukyan D., Pfeiler S., Goosmann C., Brinkmann V., Lorenz M., Bidzhekov K., Khandagale A.B. (2010). Reciprocal Coupling of Coagulation and Innate Immunity via Neutrophil Serine Proteases. Nat. Med..

[13] Mori Y., Yamaguchi M., Terao Y., Hamada S., Ooshima T., Kawabata S. (2012). α-Enolase of Streptococcus Pneumoniae Induces Formation of Neutrophil Extracellular Traps. J. Biol. Chem..

[14] Clark S.R., Ma A.C., Tavener S.A., McDonald B., Goodarzi Z., Kelly M.M., Patel K.D., Chakrabarti S., McAvoy E., Sinclair G.D. (2007). Platelet TLR4 Activates Neutrophil Extracellular Traps to Ensnare Bacteria in Septic Blood. Nat. Med..

[15] Kenny E.F., Herzig A., Krüger R., Muth A., Mondal S., Thompson P.R., Brinkmann V., von Bernuth H., Zychlinsky A. (2017). Diverse Stimuli Engage Different Neutrophil Extracellular Trap Pathways. eLife.

[16] Brinkmann V., Zychlinsky A. (2012). Neutrophil Extracellular Traps: Is Immunity the Second Function of Chromatin?. J. Cell Biol..

[17] Cassatella M.A., Östberg N.K., Tamassia N., Soehnlein O. (2019). Biological Roles of Neutrophil-Derived Granule Proteins and Cytokines. Trends Immunol..

[18] Cowland J.B., Borregaard N. (2016). Granulopoiesis and Granules of Human Neutrophils. Immunol. Rev..

[19] Silvestre-Roig C., Fridlender Z.G., Glogauer M., Scapini P. (2019). Neutrophil Diversity in Health and Disease. Trends Immunol..

[20] Sagiv J.Y., Michaeli J., Assi S., Mishalian I., Kisos H., Levy L., Damti P., Lumbroso D., Polyansky L., Sionov R.V. (2015). Phenotypic Diversity and Plasticity in Circulating Neutrophil Subpopulations in Cancer. Cell Rep..

[21] Hidalgo A., Libby P., Soehnlein O., Aramburu I.V., Papayannopoulos V., Silvestre-Roig C. (2021). Neutrophil Extracellular Traps: From Physiology to Pathology. Cardiovasc. Res..

[22] Mutua V., Gershwin L.J. (2021). A Review of Neutrophil Extracellular Traps (NETs) in Disease: Potential Anti-NETs Therapeutics. Clinic. Rev. Allergy Immunol..

[23] Manda A., Pruchniak M.P., Araźna M., Demkow U.A. (2014). Neutrophil Extracellular Traps in Physiology and Pathology. Cent.-Eur. J. Immunol..

[24] Carminita E., Crescence L., Brouilly N., Altié A., Panicot-Dubois L., Dubois C. (2021). DNAse-Dependent, NET-Independent Pathway of Thrombus Formation In Vivo. Proc. Natl. Acad. Sci. USA.

[25] Jagadeeswaran P., Cooley B.C., Gross P.L., Mackman N. (2016). Animal Models of Thrombosis From Zebrafish to Nonhuman Primates: Use in the Elucidation of New Pathologic Pathways and the Development of Antithrombotic Drugs. Circ. Res..

[26] Fish R.J., Freire C., Di Sanza C., Neerman-Arbez M. (2021). Venous Thrombosis and Thrombocyte Activity in Zebrafish Models of Quantitative and Qualitative Fibrinogen Disorders. Int. J. Mol. Sci..

[27] Lee I.-J., Yang Y.-C., Hsu J.-W., Chang W.-T., Chuang Y.-J., Liau I. (2017). Zebrafish Model of Photochemical Thrombosis for Translational Research and Thrombolytic Screening In Vivo. J. Biophotonics.

[28] Dubois C., Panicot-Dubois L., Merrill-Skoloff G., Furie B., Furie B.C. (2006). Glycoprotein VI–Dependent and –Independent Pathways of Thrombus Formation In Vivo. Blood.

[29] Denis C., Methia N., Frenette P.S., Rayburn H., Ullman-Culleré M., Hynes R.O., Wagner D.D. (1998). A Mouse Model of Severe von Willebrand Disease: Defects in Hemostasis and Thrombosis. Proc. Natl. Acad. Sci. USA.

[30] Falati S., Patil S., Gross P.L., Stapleton M., Merrill-Skoloff G., Barrett N.E., Pixton K.L., Weiler H., Cooley B., Newman D.K. (2006). Platelet PECAM-1 Inhibits Thrombus Formation In Vivo. Blood.

[31] Mangin P., Yap C.L., Nonne C., Sturgeon S.A., Goncalves I., Yuan Y., Schoenwaelder S.M., Wright C.E., Lanza F., Jackson S.P. (2006). Thrombin Overcomes the Thrombosis Defect Associated with Platelet GPVI/FcRγ Deficiency. Blood.

[32] Wang X., Smith P.L., Hsu M.-Y., Tamasi J.A., Bird E., Schumacher W.A. (2007). Deficiency in Thrombin-Activatable Fibrinolysis Inhibitor (TAFI) Protected Mice from Ferric Chloride-Induced Vena Cava Thrombosis. J. Thromb. Thrombolysis.

[33] Boulaftali Y., Lamrani L., Rouzaud M.C., Loyau S., Jandrot-Perrus M., Bouton M.C., Ho-Tin-Noé B. (2012). The mouse dorsal skinfold chamber as a model for the study of thrombolysis by intravital microscopy. Thromb. Haemost..

[34] Nayak V.K., Deschler D.G. (2005). Clopidogrel Use for Reducing the Rate of Thrombosis in a Rat Model of Microarterial Anastomosis. Arch. Otolaryngol. Head Neck Surg..

[35] Bang J., Jeon W.K. (2020). Mumefural Improves Blood Flow in a Rat Model of FeCl3-Induced Arterial Thrombosis. Nutrients.

[36] Liu H., Hou J., Hu S., Du X., Fang Y., Jia H., Feng L., Zhang L., Du J., Zhao Q. (2014). A Rabbit Model of Spontaneous Thrombosis Induced by Lipopolysaccharide. J. Atheroscler. Thromb..

[37] Costa A.F., Gamermann P.W., Picon P.X., Mosmann M.P., Kettlun A.M., Valenzuela M.A., Sarkis J.J.F., Battastini A.M.O., Picon P.D. (2004). Intravenous Apyrase Administration Reduces Arterial Thrombosis in a Rabbit Model of Endothelial Denudation In Vivo. Blood Coagul. Fibrinolysis.

[38] Shinozawa E., Kawamura M. (2018). Anti-Thrombotic Effect of a Factor Xa Inhibitor TAK-442 in a Rabbit Model of Arteriovenous Shunt Thrombosis Stimulated with Tissue Factor. BMC Res. Notes.

[39] Jankowski M., Vreys I., Wittevrongel C., Boon D., Vermylen J., Hoylaerts M.F., Arnout J. (2003). Thrombogenicity of Β2-Glycoprotein I–Dependent Antiphospholipid Antibodies in a Photochemically Induced Thrombosis Model in the Hamster. Blood.

[40] Nemmar A., Hoylaerts M.F., Hoet P.H.M., Dinsdale D., Smith T., Xu H., Vermylen J., Nemery B. (2002). Ultrafine Particles Affect Experimental Thrombosis in an In Vivo Hamster Model. Am. J. Respir. Crit. Care Med..

[41] Takiguchi Y., Hirata Y., Wada K., Nakashima M. (1992). Arterial Thrombosis Model with Photochemical Reaction in Guinea-Pig and Its Property. Thromb. Res..

[42] Moriguchi A., Aoki T., Mihara K., Tojo N., Matsuoka N., Mutoh S. (2004). Antithrombotic Effects of FK419, a Novel Nonpeptide Platelet GPIIb/IIIa Antagonist, in a Guinea Pig Photochemically Induced Middle Cerebral Artery Thrombosis Model: Comparison with Ozagrel and Argatroban. J. Pharmacol. Exp. Ther..

[43] Hosaka J., Roy S., Kvernebo K., Enge I., Laerum F. (1996). Induced Thrombosis in the Pig Inferior Vena Cava: A Model of Deep Venous Thrombosis. J. Vasc. Interv. Radiol..

[44] Kai L., Jiaxiang M., Xinxin F., Baochen L., Weiwei D., Xingjiang W., Shuofei Y., Jieshou L. (2015). Establishment of Mesenteric Venous Thrombosis in a Porcine Model Using a Transhepatic Endovascular Approach. Thromb. Res..

[45] Dogné J.-M., Rolin S., Pétein M., Tchana-Sato V., Ghuysen A., Lambermont B., Hanson J., Magis D., Segers P., Pirotte B. (2005). Characterization of an Original Model of Myocardial Infarction Provoked by Coronary Artery Thrombosis Induced by Ferric Chloride in Pig. Thromb. Res..

[46] Frisbie J.H. (2005). An Animal Model for Venous Thrombosis and Spontaneous Pulmonary Embolism. Spinal Cord.

[47] Badylak S.F., Poehlman E., Williams C., Klabunde R.E., Turek J., William S. (1988). Simple Canine Model of Arterial Thrombosis with Endothelial Injury Suitable for Investigation of Thrombolytic Agent. J. Pharmacol. Methods.

[48] Björkman J.-A.E., Abrahamsson T.I., Nerme V.K., Mattsson C.J. (2005). Inhibition of Carboxypeptidase U (TAFIa) Activity Improves Rt-PA Induced Thrombolysis in a Dog Model of Coronary Artery Thrombosis. Thromb. Res..

[49] Matafonov A., Leung P.Y., Gailani A.E., Grach S.L., Puy C., Cheng Q., Sun M., McCarty O.J.T., Tucker E.I., Kataoka H. (2014). Factor XII Inhibition Reduces Thrombus Formation in a Primate Thrombosis Model. Blood.

[50] Young W.B., Mordenti J., Torkelson S., Shrader W.D., Kolesnikov A., Rai R., Liu L., Hu H., Leahy E.M., Green M.J. (2006). Factor VIIa Inhibitors: Chemical Optimization, Preclinical Pharmacokinetics, Pharmacodynamics, and Efficacy in an Arterial Baboon Thrombosis Model. Bioorgan. Med. Chem. Lett..

[51] Tang Z., Kattula S., Holle L.A., Cooley B.C., Lin F., Wolberg A.S. (2020). Factor XIII Deficiency Does Not Prevent FeCl_3_-induced Carotid Artery Thrombus Formation in Mice. Res. Pract. Thromb. Haemost..

[52] Cleuren A.C.A., van Vlijmen B.J.M., Reitsma P.H. (2007). Transgenic Mouse Models of Venous Thrombosis: Fulfilling the Expectations?. Semin. Thromb. Hemost..

[53] Grover S.P., Mackman N. (2020). How Useful Are Ferric Chloride Models of Arterial Thrombosis?. Platelets.

[54] Denis C.V., Dubois C., Brass L.F., Heemskerk J.W.M., Lenting P.J. (2011). Biorheology Subcommittee of the SSC of the ISTH Towards Standardization of in Vivo Thrombosis Studies in Mice: Standardization of Thrombosis Studies. J. Thromb. Haemost..

[55] Barr J.D., Chauhan A.K., Schaeffer G.V., Hansen J.K., Motto D.G. (2013). Red Blood Cells Mediate the Onset of Thrombosis in the Ferric Chloride Murine Model. Blood.

[56] Eckly A., Hechler B., Freund M., Zerr M., Cazenave J.-P., Lanza F., Mangin P.H., Gachet C. (2011). Mechanisms Underlying FeCl_3_-Induced Arterial Thrombosis. J. Thromb. Haemost..

[57] Grambow E., Leppin C., Leppin K., Kundt G., Klar E., Frank M., Vollmar B. (2017). The effects of hydrogen sulfide on platelet-leukocyte aggregation and microvascular thrombolysis. Platelets.

[58] Massberg S., Gawaz M., Grüner S., Schulte V., Konrad I., Zohlnhöfer D., Heinzmann U., Nieswandt B. (2003). A Crucial Role of Glycoprotein VI for Platelet Recruitment to the Injured Arterial Wall In Vivo. J. Exp. Med..

[59] Nechipurenko D.Y., Receveur N., Yakimenko A.O., Shepelyuk T.O., Yakusheva A.A., Kerimov R.R., Obydennyy S.I., Eckly A., Léon C., Gachet C. (2019). Clot Contraction Drives the Translocation of Procoagulant Platelets to Thrombus Surface. Arterioscler. Thromb. Vasc. Biol..

[60] Faraday N., Schunke K., Saleem S., Fu J., Wang B., Zhang J., Morrell C., Dore S. (2013). Cathepsin G-Dependent Modulation of Platelet Thrombus Formation In Vivo by Blood Neutrophils. PLoS ONE.

[61] Leal A.C., Mizurini D.M., Gomes T., Rochael N.C., Saraiva E.M., Dias M.S., Werneck C.C., Sielski M.S., Vicente C.P., Monteiro R.Q. (2017). Tumor-Derived Exosomes Induce the Formation of Neutrophil Extracellular Traps: Implications For The Establishment of Cancer-Associated Thrombosis. Sci. Rep..

[62] Rosen E.D., Raymond S., Zollman A., Noria F., Sandoval-Cooper M., Shulman A., Merz J.L., Castellino F.J. (2001). Laser-Induced Noninvasive Vascular Injury Models in Mice Generate Platelet- and Coagulation-Dependent Thrombi. Am. J. Pathol..

[63] Pérez P., Alarcón M., Fuentes E., Palomo I. (2014). Thrombus Formation Induced by Laser in a Mouse Model. Exp. Ther. Med..

[64] Jin H., Gebska M.A., Blokhin I.O., Wilson K.M., Ketsawatsomkron P., Chauhan A.K., Keen H.L., Sigmund C.D., Lentz S.R. (2015). Endothelial PPAR-γ Protects Against Vascular Thrombosis by Downregulating P-Selectin Expression. Arterioscler. Thromb. Vasc. Biol..

[65] Hirata Y., Umemura K., Kondoh K., Uematsu T., Nakashima M. (1994). Experimental Intimal Thickening Studies Using the Photochemically Induced Thrombosis Model in the Guinea-Pig Femoral Artery. Atherosclerosis.

[66] Weyand A.C., Shavit J.A. (2014). Zebrafish as a Model System for the Study of Hemostasis and Thrombosis. Curr. Opin. Hematol..

[67] Marjoram R.J., Li Z., He L., Tollefsen D.M., Kunicki T.J., Dickeson S.K., Santoro S.A., Zutter M.M. (2014). A2β1 Integrin, GPVI Receptor, and Common FcRγ Chain on Mouse Platelets Mediate Distinct Responses to Collagen in Models of Thrombosis. PLoS ONE.

[68] Kleinschnitz C., Braeuninger S., Pham M., Austinat M., Nölte I., Renné T., Nieswandt B., Bendszus M., Stoll G. (2008). Blocking of Platelets or Intrinsic Coagulation Pathway–Driven Thrombosis Does Not Prevent Cerebral Infarctions Induced by Photothrombosis. Stroke.

[69] Day S.M., Reeve J.L., Pedersen B., Farris D.M., Myers D.D., Im M., Wakefield T.W., Mackman N., Fay W.P. (2005). Macrovascular Thrombosis Is Driven by Tissue Factor Derived Primarily from the Blood Vessel Wall. Blood.

[70] Shimazawa M., Kondo K., Hara H., Nakashima M., Umemura K. (2005). Sulfatides, L- and P-Selectin Ligands, Exacerbate the Intimal Hyperplasia Occurring after Endothelial Injury. Eur. J. Pharmacol..

[71] Furie B. (2005). Thrombus Formation In Vivo. J. Clin. Investig..

[72] Grover S.P., Bendapudi P.K., Yang M., Merrill-Skoloff G., Govindarajan V., Mitrophanov A.Y., Flaumenhaft R. (2020). Injury Measurements Improve Interpretation of Thrombus Formation Data in the Cremaster Arteriole Laser-Induced Injury Model of Thrombosis. J. Thromb. Haemost..

[73] Atkinson B.T., Jasuja R., Chen V.M., Nandivada P., Furie B., Furie B.C. (2010). Laser-Induced Endothelial Cell Activation Supports Fibrin Formation. Blood.

[74] Jasuja R., Furie B., Furie B.C. (2010). Endothelium-Derived but Not Platelet-Derived Protein Disulfide Isomerase Is Required for Thrombus Formation In Vivo. Blood.

[75] Passam F.H., Lin L., Gopal S., Stopa J.D., Bellido-Martin L., Huang M., Furie B.C., Furie B. (2015). Both Platelet- and Endothelial Cell–Derived ERp5 Support Thrombus Formation in a Laser-Induced Mouse Model of Thrombosis. Blood.

[76] Dubois C., Panicot-Dubois L., Gainor J.F., Furie B.C., Furie B. (2007). Thrombin-Initiated Platelet Activation In Vivo Is VWF Independent during Thrombus Formation in a Laser Injury Model. J. Clin. Investig..

[77] Chou J., Mackman N., Merrill-Skoloff G., Pedersen B., Furie B.C., Furie B. (2004). Hematopoietic Cell-Derived Microparticle Tissue Factor Contributes to Fibrin Formation during Thrombus Propagation. Blood.

[78] Gross P.L., Furie B.C., Merrill-Skoloff G., Chou J., Furie B. (2005). Leukocyte-versus Microparticle-Mediated Tissue Factor Transfer during Arteriolar Thrombus Development. J. Leukoc. Biol..

[79] Vandendries E.R., Hamilton J.R., Coughlin S.R., Furie B., Furie B.C. (2007). Par4 Is Required for Platelet Thrombus Propagation but Not Fibrin Generation in a Mouse Model of Thrombosis. Proc. Natl. Acad. Sci. USA.

[80] Brill A., Fuchs T.A., Chauhan A.K., Yang J.J., De Meyer S.F., Köllnberger M., Wakefield T.W., Lämmle B., Massberg S., Wagner D.D. (2011). Von Willebrand Factor–Mediated Platelet Adhesion Is Critical for Deep Vein Thrombosis in Mouse Models. Blood.

[81] DeRoo E., Martinod K., Cherpokova D., Fuchs T., Cifuni S., Chu L., Staudinger C., Wagner D.D. (2021). The Role of Platelets in Thrombus Fibrosis and Vessel Wall Remodeling after Venous Thrombosis. J. Thromb. Haemost..

[82] Myers D.D., Hawley A.E., Farris D.M., Wrobleski S.K., Thanaporn P., Schaub R.G., Wagner D.D., Kumar A., Wakefield T.W. (2003). P-Selectin and Leukocyte Microparticles Are Associated with Venous Thrombogenesis. J. Vasc. Surg..

[83] Martinod K., Demers M., Fuchs T.A., Wong S.L., Brill A., Gallant M., Hu J., Wang Y., Wagner D.D. (2013). Neutrophil Histone Modification by Peptidylarginine Deiminase 4 Is Critical for Deep Vein Thrombosis in Mice. Proc. Natl. Acad. Sci. USA.

[84] Martinod K., Witsch T., Farley K., Gallant M., Remold-O’Donnell E., Wagner D.D. (2016). Neutrophil Elastase-Deficient Mice Form Neutrophil Extracellular Traps in an Experimental Model of Deep Vein Thrombosis. J. Thromb. Haemost..

[85] Czaikoski P.G., Mota J.M.S.C., Nascimento D.C., Sônego F., Castanheira F.V.E.S., Melo P.H., Scortegagna G.T., Silva R.L., Barroso-Sousa R., Souto F.O. (2016). Neutrophil Extracellular Traps Induce Organ Damage during Experimental and Clinical Sepsis. PLoS ONE.

[86] Kumar S., Payal N., Srivastava V.K., Kaushik S., Saxena J., Jyoti A. (2021). Neutrophil Extracellular Traps and Organ Dysfunction in Sepsis. Clin. Chim. Acta.

[87] Zou Y., Chen X., Xiao J., Zhou D.B., Lu X.X., Li W., Xie B., Kuang X., Chen Q. (2018). Neutrophil Extracellular Traps Promote Lipopolysaccharide-Induced Airway Inflammation and Mucus Hypersecretion in Mice. Oncotarget.

[88] Thomas G.M., Carbo C., Curtis B.R., Martinod K., Mazo I.B., Schatzberg D., Cifuni S.M., Fuchs T.A., von Andrian U.H., Hartwig J.H. (2012). Extracellular DNA Traps Are Associated with the Pathogenesis of TRALI in Humans and Mice. Blood.

[89] Jarrot P.-A., Tellier E., Plantureux L., Crescence L., Robert S., Chareyre C., Daniel L., Secq V., Garcia S., Dignat-George F. (2019). Neutrophil Extracellular Traps Are Associated with the Pathogenesis of Diffuse Alveolar Hemorrhage in Murine Lupus. J. Autoimmun..

[90] Ho A.-S., Chen C.-H., Cheng C.-C., Wang C.-C., Lin H.-C., Luo T.-Y., Lien G.-S., Chang J. (2014). Neutrophil Elastase as a Diagnostic Marker and Therapeutic Target in Colorectal Cancers. Oncotarget.

[91] Kolodziej A.R., Abo-Aly M., Elsawalhy E., Campbell C., Ziada K.M., Abdel-Latif A. (2019). Prognostic Role of Elevated Myeloperoxidase in Patients with Acute Coronary Syndrome: A Systemic Review and Meta-Analysis. Mediat. Inflamm..

[92] Mauracher L.-M., Posch F., Martinod K., Grilz E., Däullary T., Hell L., Brostjan C., Zielinski C., Ay C., Wagner D.D. (2018). Citrullinated Histone H3, a Biomarker of Neutrophil Extracellular Trap Formation, Predicts the Risk of Venous Thromboembolism in Cancer Patients. J. Thromb. Haemost..

[93] Vallés J., Lago A., Santos M.T., Latorre A.M., Tembl J., Salom J., Nieves C., Moscardó A. (2017). Neutrophil Extracellular Traps Are Increased in Patients with Acute Ischemic Stroke: Prognostic Significance. Thromb. Haemost..

[94] Diaz J.A., Fuchs T.A., Jackson T.O., Kremer Hovinga J.A., Lämmle B., Henke P.K., Myers D.D., Wagner D.D., Wakefield T.W. (2013). Plasma DNA Is Elevated in Patients with Deep Vein Thrombosis. J. Vasc. Surg. Venous Lymphat. Disord..

[95] Andreas M., Sherin A., Thomas S., Thomas H., Johannes J., Adelheid P., Daniel S., Daniela L., Christine B., Andreas K. (2015). Coronary Neutrophil Extracellular Trap Burden and Deoxyribonuclease Activity in ST-Elevation Acute Coronary Syndrome Are Predictors of ST-Segment Resolution and Infarct Size. Circ. Res..

[96] Zuo Y., Yalavarthi S., Shi H., Gockman K., Zuo M., Madison J.A., Blair C.N., Weber A., Barnes B.J., Egeblad M. (2020). Neutrophil Extracellular Traps in COVID-19. JCI Insight.

[97] Hodson M.E., Shah P.L. (1995). DNase Trials in Cystic Fibrosis. Eur. Respir. J..

[98] Lauková L., Konečná B., Janovičová Ľ., Vlková B., Celec P. (2020). Deoxyribonucleases and Their Applications in Biomedicine. Biomolecules.

